# Transfusion‐related alpha‐gal syndrome: Two new cases expanding the demographic and geographic spectrum, and evidence of a diagnostic gap in allergic transfusion reaction evaluation

**DOI:** 10.1111/trf.70196

**Published:** 2026-03-28

**Authors:** Mackenzie Foster, Miriam Brown, Angela Mueller, Toufik Tahiri, Kaycie Atchison, Deva Sharma, Cosby A. Stone, Jonathan Tucci, Garrett S. Booth, Jeremy W. Jacobs

**Affiliations:** ^1^ Department of Pathology, Microbiology, and Immunology Vanderbilt University Medical Center Nashville Tennessee USA; ^2^ Quality, Safety, and Risk Prevention Vanderbilt University Medical Center Nashville Tennessee USA; ^3^ Division of Hematology/Oncology, Department of Medicine Vanderbilt University Medical Center Nashville Tennessee USA; ^4^ Division of Allergy, Pulmonary and Critical Care Medicine, Department of Medicine Vanderbilt University Medical Center Nashville Tennessee USA

**Keywords:** allergy, alpha‐gal, blood components, blood transfusion, hemovigilance, transfusion reactions

## Abstract

**Background:**

Transfusion‐related alpha‐gal syndrome (TRAGS) has recently been proposed as a cause of allergic transfusion reactions (ATRs) in which alpha‐gal‐specific IgE in sensitized group O (or potentially group A) recipients reacts with epitopes on group B or AB plasma‐containing components. Fewer than 10 cases have been reported, all from the Northeast and mid‐Atlantic United States, and alpha‐gal–specific ATR evaluation practices are unstudied.

**Study Design and Methods:**

Two patients with ATRs consistent with TRAGS at a large academic medical center in Nashville, Tennessee are reported alongside a 5‐year retrospective cohort analysis of group O and A recipients experiencing ATRs following transfusion of group B or AB plasma‐containing products. Alpha‐gal IgE testing, AGS diagnosis documentation, and documented consideration of IgA deficiency were assessed for each qualifying reaction.

**Results:**

Both index patients had pre‐existing AGS diagnoses unrecognized at component selection; one was a 42‐year‐old female and the second an 83‐year‐old male. Among 50 qualifying ATRs in 44 patients, including 13 severe or anaphylactic reactions, alpha‐gal IgE testing was not performed for any event, and no patient had a documented AGS diagnosis. IgA deficiency was considered in eight patients (18%), yielding no diagnoses.

**Conclusion:**

TRAGS occurs in the tick‐endemic southeastern United States across a broader demographic range than previously recognized. IgA deficiency, present in <0.3% of the population, was considered in 18% of qualifying patients while alpha‐gal sensitization, affecting 20%–30% in this region, was investigated in none. Integration of AGS history into pre‐transfusion risk assessment and ATR evaluation protocols is warranted.

**Abbreviations**
ATR: allergic transfusion reaction
AGS: alpha‐gal syndrome
BEST: biomedical excellence for safer transfusion
CDC: Centers for Disease Control and Prevention
DAT: direct antiglobulin test
EMR: electronic medical record
IgE: immunoglobulin E
RBC: red blood cell
SICU: surgical intensive care unit
TRAGS: transfusion‐related alpha‐gal syndrome
VUMC: Vanderbilt University Medical Center

## 1 INTRODUCTION

Allergic transfusion reactions (ATRs) are among the most frequently reported acute adverse events following transfusion and occur most commonly with plasma‐containing blood components, including platelet and plasma products.^1^ Despite their frequency, the inciting antigen responsible for an ATR is identified in only a minority of cases, limiting opportunities for targeted prevention and informed component selection for future transfusion episodes.^1^, ^2^, ^3^ Recent reports have drawn attention to alpha‐gal syndrome (AGS) as a potential and previously under‐recognized contributor to severe ATRs in a specific clinical context, with transfusion of group B or AB plasma‐containing components (i.e., plasma, platelets, and potentially cryoprecipitate) to group O recipients.^4^, ^5^, ^6^

AGS is an immunoglobulin E (IgE)–mediated allergy to galactose‐α‐1,3‐galactose (alpha‐gal), a carbohydrate epitope expressed on cells of most non‐primate mammals.^7^, ^8^ First described in 2009, AGS in the United States is primarily associated with bites from the lone star tick (*Amblyomma americanum*), although emerging evidence suggests that other tick species, including *Ixodes scapularis*, may also trigger AGS in some settings^9^; other tick species have been implicated globally.^8^, ^10^ Tick saliva has been shown to contain alpha‐gal‐bearing molecules, and the inflammatory/immunomodulatory cutaneous microenvironment at the bite site appears to favor a Th2/IgE‐skewed immune response to alpha‐gal.^10^ The condition classically manifests as delayed hypersensitivity reactions, including urticaria, angioedema, gastrointestinal symptoms, and anaphylaxis, occurring 2–6 h after ingestion of mammalian meat or mammalian‐derived products.^7^, ^8^ AGS is an emerging public health concern; the US Centers for Disease Control and Prevention (CDC) identified over 110,000 suspected cases between 2010 and 2022, with estimates of up to 450,000 individuals affected.^8^, ^11^ The geographic clustering of suspected cases broadly overlaps the established range of the lone star tick, although cases have been reported outside that range.^8^, ^9^, ^11^

The alpha‐gal epitope is structurally similar to the blood group B antigen (Galα1‐3[Fucα1,2]Gal).^12^, ^13^ This molecular resemblance raises the possibility that patients with alpha‐gal‐specific IgE may be at risk for IgE‐mediated reactions when exposed to group B antigen‐containing blood components. Individuals with blood group B or AB may have partial immunologic tolerance to alpha‐gal due to endogenous B antigen expression, conferring some protection against the development of AGS.^7^, ^14^, ^15^ Conversely, group O (and potentially group A) recipients who lack the B antigen may be particularly vulnerable to cross‐reactive IgE‐mediated reactions upon transfusion with group B or AB plasma and platelets.

To date, six patients with suspected transfusion‐related alpha‐gal syndrome (TRAGS) have been described in the literature, all from the Northeast and mid‐Atlantic United States.^4^, ^5^, ^6^ In 2023, Gilstad et al. reported three group O patients in the Washington, DC metropolitan area who experienced anaphylactic reactions after transfusion of group B plasma or platelets, one of which was fatal.^4^ Miller et al. subsequently described two additional critically ill group O patients in the same region who developed anaphylaxis following group B plasma transfusion.^5^ Most recently, Jones et al. reported a group O patient who developed anaphylactic shock after receiving a group B platelet unit, with in vitro immunologic findings suggestive of alpha‐gal IgE‐mediated pathophysiology.^6^ All previously reported patients for whom demographic data are available have been males over the age of 50. These reports prompted the Biomedical Excellence for Safer Transfusion (BEST) Collaborative to quantify the frequency of ABO‐mismatched transfusion exposures that could place patients at risk, finding that group O recipients received group B plasma and platelets for an average of 3.2% and 4.1% of respective transfusions across 14 international sites.^16^ A nationwide French hemovigilance analysis similarly identified an association between group B major ABO‐incompatible transfusions and severe allergic reactions, further supporting a potential role for alpha‐gal‐mediated mechanisms.^17^ Whereas published TRAGS case reports have thus far been limited to severe or anaphylactic presentations, the BEST Collaborative study and the French hemovigilance data suggest that alpha‐gal‐related risk may extend across a broader spectrum of allergic transfusion reactions. A 2026 scoping review of alpha‐gal sensitization and allergic blood transfusion reactions synthesized the emerging TRAGS literature and highlighted both a plausible relationship between alpha‐gal sensitization and blood group biology and the absence of evidence‐based diagnostic guidance for suspected TRAGS.^18^

Despite emerging recognition, the geographic and demographic breadth of TRAGS remains poorly characterized, and it is unknown how frequently AGS contributes to ATRs currently classified as idiopathic. To our knowledge, no study has assessed the extent to which alpha‐gal‐specific diagnostic evaluation is incorporated into the workup of ATRs in eligible patients.

Herein, we report two patients at a large academic medical center in the southeastern United States who experienced severe ATRs with clinical and serologic features consistent with TRAGS. We additionally present the results of a single‐center retrospective analysis of all group O and A recipients who experienced ATRs following exposure to group B or AB plasma‐containing components over a five‐year period, characterizing the frequency with which alpha‐gal‐specific diagnostic evaluation was pursued in this high‐risk cohort.

## 2 METHODS

### 2.1 Study design and setting

This study comprised two components: (1) case reports of two patients with severe ATRs and serologic features consistent with TRAGS, and (2) a retrospective observational analysis of all group O and A patients who experienced ATRs following exposure to group B or AB platelets or plasma at Vanderbilt University Medical Center (VUMC), Nashville, Tennessee, from January 1, 2021 through December 31, 2025. This contemporary 5‐year interval was selected to permit detailed manual review of transfusion reaction records and electronic medical record (EMR) documentation while evaluating TRAGS recognition within current institutional transfusion medicine workflows. Nashville lies within the endemic range of *A. americanum* and within one of the highest‐density US regions of suspected AGS.^11^

Suspected transfusion reactions at VUMC are reported by the treating clinical team and evaluated by the transfusion medicine service, with review in service rounds and documentation in the EMR. No prospective hemovigilance program was in place during the study period. VUMC also maintains a dedicated AGS clinical program through which affected patients may receive longitudinal care, although this infrastructure was not systematically integrated into transfusion reaction evaluation workflows.

This study was approved by the VUMC Institutional Review Board [IRB #: 260112] with waivers of informed consent and HIPAA authorization.

### 2.2 Retrospective cohort

Eligible patients were ABO group O or A recipients who experienced a reported, transfusion medicine service‐evaluated ATR during or following transfusion of at least one group B or AB plasma‐containing component (platelets and/or plasma products including fresh frozen plasma, plasma frozen within 24 h of phlebotomy, thawed plasma, or liquid plasma). The cohort included ATRs across the spectrum of reported severity, including reactions considered mild, provided they were formally reported and classified as allergic by the transfusion medicine service at the time of evaluation. Reactions classified as non‐allergic (e.g., febrile non‐hemolytic transfusion reaction, transfusion‐associated circulatory overload, transfusion‐related acute lung injury, unrelated/underlying medical condition) were not included in the review. Eligible patients were identified from transfusion service reaction records, linked to blood bank laboratory information system data to confirm recipient ABO type, component ABO group, and component type.

### 2.3 Data collection and AGS ascertainment

Clinical, transfusion, and allergy/immunology data were abstracted from the EMR, including reaction severity based on the clinical assessment of the transfusion medicine team at the time of the reaction, product details, premedication use, allergy history, and clinical outcomes. Because case identification depended on formal reaction reporting and transfusion medicine evaluation, bedside‐managed reactions that were not reported to the transfusion medicine service were not captured. For each patient, we assessed whether alpha‐gal‐specific IgE testing had been performed (all testing during the study period was performed at a reference laboratory) and whether AGS‐related history was documented in the EMR. AGS ascertainment was based on manual review of structured fields and free‐text EMR documentation. Specifically, we assessed structured problem lists and allergy fields for documentation of AGS or meat allergy and performed free‐text searches of the EMR for the terms “alpha‐gal,” “tick,” “IgE,” “meat allergy,” “meat,” “beef,” “pork,” “venison,” “lamb,” and “dairy.” Allergy to mammalian‐derived pharmaceuticals, biologics, or vaccines was not systematically assessed. Explicit documentation of IgA deficiency, either through formal testing or consideration as a contributor, was recorded as a comparator. Patients were classified as: *consistent with TRAGS* (alpha‐gal IgE results available in the EMR); *suspected TRAGS* (documented mammalian meat allergy without IgE results); *possible TRAGS* (documented tick bite history without IgE results or AGS diagnosis); or *unevaluated for AGS*. This study‐specific classification schema was developed for retrospective ascertainment of possible TRAGS and was not intended to be a formal adaptation of AGS surveillance case definitions. Descriptive statistics were used throughout; continuous variables are reported as means with standard deviations or medians with interquartile ranges, and categorical variables as frequencies and percentages.

## 3 RESULTS

The two cases presented below were identified independently in clinical practice and prompted the institutional review described herein. They are therefore presented separately and are not included in the retrospective cohort.

### 3.1 Case 1

The patient was a 42‐year‐old female, blood group O, RhD‐positive, with a history of penetrating Crohn's disease requiring prior endoscopic dilations and small bowel resection(s), and documented tick bite history with a known pre‐existing diagnosis of AGS (Table 1). She had a mild food‐triggered AGS phenotype characterized by gastrointestinal symptoms without anaphylaxis, and reported no reactions to dairy products, gelatin, or heparin. The overlap of AGS‐related gastrointestinal symptoms with her underlying Crohn's disease may have contributed to underrecognition of the full significance of her alpha‐gal sensitization in the perioperative context. She presented for elective right ileocolectomy and small bowel resection. Following the procedure, she was transferred to the surgical intensive care unit (SICU) for management of acute blood loss anemia, at which point transfusion of blood products was initiated.

**TABLE 1:** Clinical and laboratory characteristics of the two index cases of transfusion‐related alpha‐gal syndrome.

Characteristic	Case 1	Case 2
Patient characteristics		
Age/sex	42 years/female	83 years/male
Blood group	O, RhD‐positive	O, RhD‐positive
Relevant medical history	Penetrating Crohn's disease; prior endoscopic dilations and small bowel resections	Atrial fibrillation (apixaban); coronary artery disease (clopidogrel); ground‐level fall with subdural hematoma
Pre‐existing AGS diagnosis	Yes: GI symptoms with meat ingestion without anaphylaxis; no reactions to dairy, gelatin, or heparin; documented tick bite history. Not routinely following in VUMC AGS clinic.	Yes: documented anaphylaxis to beef and pork; AGS in allergy record and problem list at admission. Not routinely following in VUMC AGS clinic.
Indication for transfusion	Acute blood loss anemia following right ileocolectomy and small bowel resection (laparoscopic converted to open)	Anticoagulant/antiplatelet reversal in setting of traumatic subdural hematoma
Transfusion details		
Component (ABO, RhD)	Thawed plasma (AB, RhD‐positive)	Pathogen reduced apheresis platelets in plasma (B, RhD‐positive)
Volume administered before reaction	~179 mL	~30 mL
Time to reaction onset from initiation	~33 min	~32 min
Premedication	None	None
Reaction		
Symptoms	Chest pain, flushing, tachycardia, urticaria, tachypnea	Dyspnea, flushing, tachycardia, tachypnea, urticaria, bronchospasm with wheezing (15 L/min O_2_ via non‐rebreather mask)
Severity classification	Severe/anaphylactic	Severe/anaphylactic
Setting	Surgical ICU	Trauma ICU
Escalation of care required	None (continuous monitoring already in place)	None (continuous monitoring already in place)
Treatment	Epinephrine IM, albuterol, corticosteroids, diphenhydramine	Epinephrine IM ×3, epinephrine infusion, diphenhydramine, famotidine, ondansetron
Symptom resolution	Yes, following treatment	Yes, gradual, over several hours
Workup and diagnosis		
Clerical check, DAT, hemolysis assessment	Unremarkable	
Serum tryptase	Not obtained	
Total IgE	Not obtained	
Allergy/immunology consultation	Not obtained	
Alpha‐gal–specific IgE (reference range ≤0.09 kU/L)	0.59 kU/L (previously measured as part of AGS evaluation)	5.82 kU/L
TRAGS classification	Consistent with TRAGS	
Outcome		
Transfusion restriction placed	Yes—group B and AB platelets and plasma restricted	
AGS documentation in EMR	Pre‐existing in allergy record and problem list	
Allergy/immunology referral	Not obtained	

Abbreviations: AGS, alpha‐gal syndrome; DAT, direct antiglobulin test; GI, gastrointestinal; IgE, immunoglobulin E; IM, intramuscular; VUMC, Vanderbilt University Medical Center.

The patient received 1 unit of group AB, RhD‐positive thawed plasma without pre‐transfusion premedication. Approximately 33 min after transfusion initiation, at which point approximately 179 mL had been administered, she developed chest pain, flushing, tachypnea, tachycardia, and urticaria; the transfusion was stopped upon recognition of the reaction. The reaction was treated with intramuscular epinephrine, inhaled albuterol, corticosteroids, and diphenhydramine. As the patient was already under continuous monitoring in the surgical intensive care unit at the time of the reaction, no escalation of care was required. Symptoms resolved following the treatment measures described. She had received one unit of group O thawed plasma and two units of group O, RhD‐positive RBCs earlier in the same admission without incident.

Transfusion reaction workup was initiated per institutional protocol. Standard transfusion reaction workup, including clerical check, direct antiglobulin test (DAT), and hemolysis assessment, was unremarkable. Serum tryptase and total IgE were not obtained. Alpha‐gal‐specific IgE, which had been measured previously as part of her AGS evaluation, was 0.59 kU/L (reference range ≤0.09 kU/L). A mammalian meat allergy panel was not performed. In the setting of a pre‐existing AGS diagnosis, the workup focused on alpha‐gal sensitization rather than broader allergy testing, and no allergy/immunology consultation was obtained during the index evaluation. The transfusion medicine service classified the reaction as anaphylactic, and the clinical presentation was consistent with TRAGS. Despite the documented AGS diagnosis in the medical record, it was not recognized as a transfusion risk factor at the time of blood product selection. A transfusion restriction was subsequently placed to avoid future administration of group B or AB platelets and plasma.

### 3.2 Case 2

The patient was an 83‐year‐old male, blood group O, RhD‐positive, with a history of atrial fibrillation on apixaban and coronary artery disease on clopidogrel (Table 1). He presented following a ground‐level fall and subsequent subdural hematoma. The patient had a pre‐existing documented allergy to beef and pork with a reaction history of anaphylaxis, consistent with a clinical diagnosis of alpha‐gal syndrome. Alpha‐gal syndrome was documented in both the allergy record and problem list at the time of presentation.

The patient received one unit of group B, RhD‐positive pathogen reduced apheresis platelets stored in plasma without pre‐transfusion premedication. Approximately 32 min after transfusion initiation, at which point approximately 30 mL had been administered, he developed dyspnea, flushing, tachycardia, and urticaria involving the abdomen, accompanied by bronchospasm with wheezing requiring supplemental oxygen at 15 L/min via non‐rebreather mask; the transfusion was stopped upon recognition of the reaction. He required three separate administrations of intramuscular epinephrine and initiation of a continuous epinephrine infusion, in addition to diphenhydramine, famotidine, and ondansetron. As the patient was already under continuous monitoring in the trauma ICU at the time of the reaction, no escalation of care was required. Symptoms gradually resolved over several hours with the epinephrine infusion, which was weaned by the following morning. There was no documented history of prior transfusion.

Standard transfusion reaction workup, including clerical check, DAT, and hemolysis assessment, was unremarkable. Alpha‐gal‐specific IgE was elevated at 5.82 kU/L (reference range ≤0.09 kU/L), and a mammalian meat allergy panel demonstrated elevated beef, pork, and lamb IgE levels of 2.19, 0.99, and 0.91 kU/L, respectively (reference range ≤0.34 kU/L for each). The reaction was classified as anaphylactic by the transfusion medicine service. Serum tryptase and total IgE were not obtained. Taken together with his pre‐existing documented history of anaphylaxis to mammalian meat, the clinical presentation was consistent with TRAGS. A transfusion restriction was placed precluding future administration of group B or AB platelets and plasma. Allergy/immunology consultation was not obtained.

### 3.3 Retrospective review

During the study period, 50 ATRs in 44 unique patients met inclusion criteria (Table 2). The cohort was predominantly male (29 of 44 unique patients [66%]), with a mean age of 57.8 years (median 63, range 12–88). Four patients were 19 years of age or younger, including two adolescents aged 12 and 13. Forty‐one reactions (82%) occurred in group O recipients and 9 (18%) in group A recipients. Thirty‐one reactions (62%) involved group B products and 19 (38%) involved group AB products. Forty‐one reactions (82%) involved platelet products and 9 (18%) involved plasma products. Ten of 50 reactions (20%), representing 7 unique patients, occurred in hematopoietic stem cell transplant recipients. All transplant donor‐recipient pairings in this subgroup were either ABO‐identical (*n* = 4 patients), autologous (*n* = 2 patients), or a group A donor to group AB recipient (*n* = 1 patient); no patient had discordant ABO/Rh typing results.

**TABLE 2:** Characteristics of all qualifying allergic transfusion reactions and unique patients identified in the retrospective cohort (January 2021–December 2025).

Characteristic	Reactions (*N* = 50)	Patients (*N* = 44)
Recipient ABO type		
Group O	41 (82%)	36 (82%)
Group A	9 (18%)	8 (18%)
Implicated component ABO group		
Group B	31 (62%)	—
Group AB	19 (38%)	—
Component type		
Platelets	41 (82%)	—
Plasma	9 (18%)	—
Reaction severity		
Mild	24 (48%)	—
Moderate	13 (26%)	—
Severe/anaphylactic	13 (26%)	—
Recurrent ATRs during study period		
Patients with ≥2 ATRs	—	5 (11.4%)
2 ATRs	—	4 (9.1%)
3 ATRs	—	1 (2.3%)
Allergy/immunology consultation		
Allergy/immunology consulted in association with ATR^a^	5 (10%)	3 (7%)
Alpha‐gal–specific evaluation		
Alpha‐gal IgE testing performed as part of ATR workup	0 (0%)	0 (0%)
AGS diagnosis documented in EMR	0 (0%)	0 (0%)
Tick bite history documented in EMR at time of ATR	4 (8%)	3 (7%)
Mammalian meat allergy documented in clinical notes^b^	1 (2%)	1 (2%)
Prior alpha‐gal evaluation noted (no IgE values in EMR)^c^	1 (2%)	1 (2%)
TRAGS classification		
Consistent with TRAGS (alpha‐gal IgE results in EMR)	0 (0%)	0 (0%)
Suspected TRAGS (documented mammalian meat allergy, no IgE)	1 (2%)	1 (2%)
Possible TRAGS (tick bite history, no IgE or AGS diagnosis)	4 (8%)	3 (7%)
Unevaluated for AGS	45 (90%)	40 (91%)

*Note*: Percentages may not sum to 100% due to rounding.

Abbreviations: AGS, alpha‐gal syndrome; ATR, allergic transfusion reaction; FFP, fresh frozen plasma; FP24, plasma frozen within 24 h of phlebotomy; TRAGS, transfusion‐related alpha‐gal syndrome.

^a^ Allergy/immunology consultation refers to consultation obtained during the index transfusion reaction evaluation, or in the immediate follow‐up period specifically for the allergic reaction and does not exclude prior outpatient allergy evaluation.

^b^ Documented in an external clinical note (Care Everywhere, Epic); not captured in the structured allergy field of the electronic medical record.

^c^ Notation referencing a prior alpha‐gal panel with a negative result was identified in a provider note; no quantitative IgE values were available in the EMR and no alpha‐gal evaluation was performed in association with the qualifying reaction.

Reaction severity ranged from mild to severe/anaphylactic. By institutional classification, 24 reactions (48%) were mild, 13 (26%) were moderate, and 13 (26%) were severe/anaphylactic. Twelve of the severe/anaphylactic reactions occurred in group O recipients, while 1 occurred in a group A recipient; 9 (69%) involved group B components and 4 (31%) involved group AB components. Five patients (11%) experienced recurrent ATRs during the study period, for a total of 11 reactions. One 41‐year‐old group O male with decompensated cirrhosis, but without documented IgA deficiency, experienced two separate anaphylactic reactions on the same calendar day following sequential exposure to group AB plasma and a group B platelet unit. While this patient was particularly notable, the retrospective design does not allow distinction between unrecognized TRAGS and a broader patient‐specific predisposition to recurrent allergic transfusion reactions.

Alpha‐gal‐specific IgE evaluation was not performed as part of the transfusion reaction workup for any of the 50 qualifying reaction events, and no patient in the cohort had an AGS diagnosis documented in the EMR at the time of their ATR. In contrast, IgA deficiency was explicitly evaluated or documented as a consideration in 8 of 44 patients (18%), returning normal or elevated results in all cases.

Several additional features supportive of possible or suspected TRAGS were identified on medical record review. Three patients (7%) representing four qualifying reactions had documentation of prior tick bite exposure and were classified as possible TRAGS. The three patients were a 61‐year‐old male group A recipient (mild ATR, group B platelets), a 68‐year‐old male group O recipient (mild ATR, group AB platelets), and a 71‐year‐old male group O recipient who experienced two mild ATRs following group B platelet transfusions 1 month apart. One additional patient (2%), an 82‐year‐old, group O female who experienced an anaphylactic reaction to group B platelets, was found to have a beef allergy documented in an external clinical note at the time of a qualifying anaphylactic reaction, meeting criteria for suspected TRAGS. This allergy was absent from the structured EMR allergy field and no alpha‐gal evaluation was pursued, illustrating how relevant clinical information may escape routine reaction evaluation.

Applying the prespecified classification schema, 1 patient (2%) was classified as suspected TRAGS, 3 (7%) as possible TRAGS, and the remaining 40 (91%) as unevaluated for AGS. No patient in the cohort was classifiable as consistent with TRAGS, as no alpha‐gal IgE testing was performed in association with any qualifying reaction.

## 4 DISCUSSION

We report two patients with reactions serologically consistent with TRAGS at a large academic medical center in Nashville, Tennessee, expanding the recognized geographic range of this entity beyond the Northeast and mid‐Atlantic United States to the AGS‐endemic southeastern region and adding to its described clinical spectrum. All previously reported patients for whom demographic data are available have been males over the age of 50 who received group B platelets or plasma (Table 3). Our cases expand this currently reported profile; Case 1 was a 42‐year‐old female who developed anaphylaxis after transfusion of group AB plasma and had an alpha‐gal‐specific IgE level of 0.59 kU/L. Case 2 was an 83‐year‐old male who developed anaphylaxis after transfusion of group B platelets whose alpha‐gal‐specific IgE of 5.82 kU/L is among the highest reported in association with TRAGS. However, alpha‐gal–specific IgE concentrations should be interpreted cautiously because titers may fluctuate over time, often decline with avoidance of recurrent tick bites, and do not correlate in a simple linear fashion with reaction severity.^19^, ^20^ Together, these cases suggest that TRAGS is not confined to the limited demographic and exposure pattern reflected in the small number of published reports and may occur across a broad range of patient characteristics and alpha‐gal sensitization levels.

**TABLE 3:** Summary of all reported cases of transfusion‐related alpha‐gal syndrome (TRAGS).

Case	Author, year	Institution/location	Age	Sex	Recipient ABO	Component (ABO)	Alpha‐gal IgE (kU/L)^a^	Tryptase	Outcome	Notes
1	Gilstad, 2023^4^	MedStar Georgetown University Hospital, Washington, DC	NR	NR	O	Thawed plasma (B)	Not tested	28.9 μg/L (↑)	Fatal (intraoperative)	Liver transplant; allergy/anaphylaxis; two prior transfusions with RBCs without reaction.
2	Gilstad, 2023^4^	MedStar Washington Hospital Center, Washington, DC	59	M	O	Thawed plasma (B), ×3 separate admissions	0.72	NR	Survived	Cirrhosis; tick bites; GI complaints; history of 42 previous blood component transfusions and severe allergic transfusion reactions to three products
3	Gilstad, 2023^4^	MedStar Georgetown University Hospital, Washington, DC	NR	M	O	Psoralen‐treated apheresis platelets (B)	3.04	NR	Died day 25 (Klebsiella pneumonia, PTLD)^b^	Traumatic ICH; Crohn's; kidney transplant; cat/dog/horse allergies; tick bites
4	Miller, 2024^5^	NIH Clinical Center, Bethesda, MD	64	M	O	FFP (B)	1.96	54.1 ng/mL (↑)	Died (sepsis, underlying causes)^b^	Heart transplant on ECMO; plasma exchange; from Texas; no known allergies
5	Miller, 2024^5^	MedStar Washington Hospital Center, Washington, DC	57	M	O	FFP (B), ×2 units	10.3	5.7 ng/mL (neg)^c^	Survived	Melanoma; neurofibromatosis type 1; abdominal resection; tick bites; from NC
6	Jones, 2025^6^	Dartmouth Hitchcock Medical Center, Lebanon, NH	76	M	O	Apheresis platelets (B)	1.49	27.4 ng/mL (↑)	Survived (d/c POD 4)	CABG + AVR; tick bites; no known allergies; iBAT positive for alpha‐gal and B antigen
7	Present report	Vanderbilt University Medical Center, Nashville, TN	42	F	O	Thawed plasma (AB)	0.59	Not obtained	Survived	Known AGS (GI phenotype; tolerates dairy, gelatin, heparin); Crohn's; SICU; received group O thawed plasma and group O RBCs earlier in same admission without incident; no prior transfusion history documented
8	Present report	Vanderbilt University Medical Center, Nashville, TN	83	M	O	Pathogen reduced apheresis platelets in plasma (B)	5.82	Not obtained	Survived	Known AGS (beef/pork anaphylaxis documented in allergy record); SDH; Trauma ICU; no prior transfusion history documented

*Note*: Cases 7 and 8 are from the present report.

Abbreviations: AGS, alpha‐gal syndrome; AVR, aortic valve replacement; CABG, coronary artery bypass graft; d/c, discharged; ECMO, extracorporeal membrane oxygenation; FFP, fresh frozen plasma; GI, gastrointestinal; iBAT, indirect basophil activation test; ICH, intracranial hemorrhage; ICU, intensive care unit; NF1, neurofibromatosis type 1; NR, not reported; POD, postoperative day; PTLD, post‐transplant lymphoproliferative disorder; RBCs, red blood cells; SDH, subdural hematoma; SICU, surgical intensive care unit; TRAGS, transfusion‐related alpha‐gal syndrome.

^a^ Reference range ≤0.09 kU/L unless otherwise specified. Cases 1–3 (Gilstad) used reference range <0.1 kU/L; Case 4 (Miller, ARUP) used reference range ≤0.09 kU/L; Case 5 (Miller, Mayo) used positive cutoff >0.70 kU/L; Case 6 (Jones) used reference range <0.10 kU/L.

^b^ Death was not attributed directly to the anaphylactic transfusion reaction; patients died of subsequent complications or underlying conditions during the same or a later hospitalization.

^c^ Tryptase was collected within 30 min of the reaction but may have been performed late; a negative result does not exclude anaphylaxis.

A distinctive and clinically important feature of both cases is that each patient carried a pre‐existing AGS diagnosis in the medical record at the time of transfusion, yet in neither case was AGS recognized as a transfusion‐relevant risk factor during component selection. To our knowledge, this represents a previously unreported scenario in the TRAGS literature and suggests a need for EMR alerts or other decision‐support tools when alpha‐gal allergy is known prior to transfusion. In Case 1, the diagnosis may have been functionally obscured by the clinical overlap between AGS‐related gastrointestinal symptoms and her underlying Crohn's disease. In Case 2, a documented history of anaphylaxis to beef and pork was recorded in both the allergy record and problem list, yet group B platelets were administered without premedication and without documented consideration of ABO‐alternative components. This case further illustrates that failure to recognize AGS may have implications beyond transfusion support alone, particularly in patients with cardiovascular disease, in whom mammalian‐derived exposures may include porcine heparin, gelatin‐based hemostatic agents, bovine pericardial patches, and bioprosthetic materials.^21^ Although such exposures were not implicated in the present reaction, they underscore the broader clinical importance of recognizing AGS across care settings. These cases show that preventing TRAGS requires more than documentation of an AGS diagnosis; clinical systems and provider awareness will need to translate that diagnosis into transfusion guidance at the point of component selection.

Case 1 illuminates an important aspect of alpha‐gal sensitization, in which the relationship between oral phenotype (i.e., food allergy symptoms) and intravenous reaction severity is not reliably predictive. The patient had a modestly elevated alpha‐gal‐specific IgE and a mild food‐triggered phenotype characterized by gastrointestinal symptoms without anaphylaxis, with tolerance of products containing lower alpha‐gal densities than red meat, including dairy, gelatin, and heparin. Despite this attenuated oral profile, she experienced severe anaphylaxis when exposed via the intravenous route, a discordance consistent with the recognized pattern that parenteral alpha‐gal exposure, by bypassing gastrointestinal degradation and delivering antigen directly into the systemic circulation, carries risk of more severe reactions in patients with mild or absent food‐triggered symptoms or asymptomatic sensitization.^19^, ^22^, ^23^, ^24^, ^25^ Clinicians should not use a mild or absent food‐triggered history to discount TRAGS risk in patients with documented alpha‐gal sensitization receiving group B or AB plasma‐containing components.

Both reactions occurred during active transfusion, with symptom onset at approximately 30 min after initiation, in notable contrast to the classic 2–6 h delayed presentation of food‐triggered AGS reactions.^7^, ^8^ This pattern mirrors the immediate hypersensitivity observed with intravenous cetuximab, a monoclonal antibody carrying alpha‐gal due to its production in a murine cell line, which produces acute anaphylaxis upon infusion in sensitized patients and represents the closest established mechanistic precedent for intravenous‐route TRAGS.^14^, ^25^ In addition to cetuximab, parenteral or procedural exposures (e.g., mammalian‐derived products such as porcine heparin, gelatin‐based hemostatic agents, bioprosthetic materials) can cause severe reactions in patients with alpha‐gal hypersensitivity.^22^, ^26^ Practically, a reaction occurring during or shortly after transfusion in a group O or A recipient receiving group B or AB components should prompt TRAGS in the differential, particularly when symptoms include urticaria, angioedema, or bronchospasm without an alternative explanation.

The absence of alpha‐gal‐specific diagnostic evaluation in the retrospective cohort was observed across all severity levels, including the 13 severe or anaphylactic reactions, and persisted even when allergy/immunology consultation was obtained, occurring in association with five reactions across three patients. A particularly important finding was that clinically relevant AGS‐related history could be present in the EMR yet remain difficult to identify during the transfusion reaction workup. In one case, a patient developed anaphylaxis while a beef allergy was documented only in an external clinical note and did not populate the structured allergy field; in another, a patient experienced recurrent reactions despite tick bite history being documented elsewhere in the record. These examples suggest that the challenge is not solely lack of awareness of AGS,^27^ but also the fragmented and inconsistently documented way in which relevant history and results may be stored in the EMR. These patterns underscore the need for formal guidance and workflow‐level support for clinicians and transfusion medicine services. Our findings are consistent with a recent scoping review of the TRAGS literature, which concluded that clinical guidance remains limited and that no validated diagnostic algorithm currently exists.^18^ That review emphasized the importance of interpreting laboratory findings in the context of clinical history and suggested that alpha‐gal‐specific IgE testing should be considered in patients with unexplained severe transfusion reactions who have a history of AGS or tick exposure. Collectively, these observations support incorporating alpha‐gal‐focused history taking and diagnostic testing into transfusion reaction evaluation pathways, particularly in endemic regions.

**FIGURE 1:**
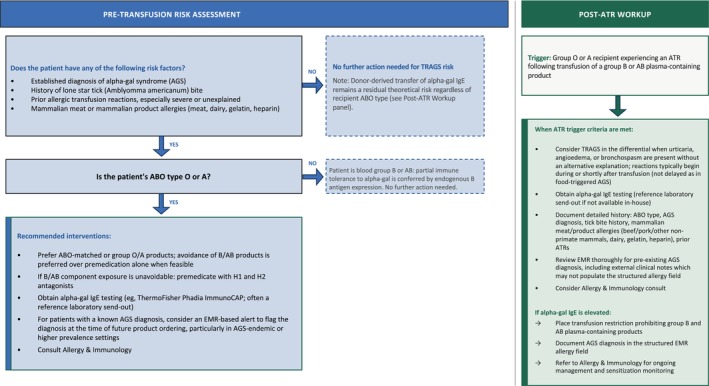
Proposed algorithm to reduce the risk of transfusion‐related alpha‐gal syndrome (TRAGS) in *Amblyomma americanum*‐endemic regions. Left panel: pre‐transfusion risk assessment for group O and A patients. Right panel: post‐reaction evaluation pathway for any qualifying allergic transfusion reaction (ATR). AGS, alpha‐gal syndrome; ATR, allergic transfusion reaction; EMR, electronic medical record; IgE, immunoglobulin E.

The BEST Collaborative has appropriately focused TRAGS risk reduction on group O‐preferential component strategies,^16^ but our data suggest that component policy alone may be insufficient, and that AGS ascertainment in pre‐transfusion evaluation and integration of known AGS diagnoses into transfusion ordering workflows represent complementary priorities, particularly in endemic regions. The magnitude of the current diagnostic gap is perhaps best illustrated by a single observation: IgA deficiency, a condition affecting fewer than 0.3% of the general population,^28^ was explicitly evaluated or documented as a consideration in 18% of patients, while alpha‐gal sensitization was investigated in none. In a region where alpha‐gal sensitization may affect 20%–30% of individuals and clinical AGS may exceed 2% of the population in endemic regions,^29^, ^30^ this disparity reflects the early stage of clinical awareness surrounding AGS as a transfusion risk factor and underscores the need for guidance to support its integration into clinical decision‐making (Figure 1).

Several limitations warrant acknowledgment. Both case reports are from a single institution and neither patient underwent IgA or haptoglobin testing. Additionally, serum tryptase was not obtained in either case; as in other reported TRAGS cases, this likely reflects the operational reality that tryptase is time‐sensitive, not always readily available during acute transfusion reaction management, and often not required for immediate clinical decision‐making.^18^ The absence of systematic alpha‐gal IgE testing in the retrospective cohort precludes definitive determination of TRAGS prevalence. Retrospective classification depends on documented history, and tick bite exposure and dietary allergy are likely underreported in the inpatient setting, meaning alpha‐gal sensitization in this cohort is probably underestimated. The retrospective cohort was limited to reactions formally reported and classified as allergic transfusion reactions by the transfusion medicine service at the time of evaluation. Accordingly, possible TRAGS‐like cases with mixed or competing features may have been missed, and mild reactions managed at the bedside without formal reporting would not have been captured. Findings may not generalize beyond high‐prevalence regions, and the absence of total transfusion denominators limits calculation of a population‐based TRAGS incidence. Additionally, indirect basophil activation testing using B antigen as the allergen, which was recently demonstrated to show positive results in a confirmed TRAGS patient,^6^ was not performed in either case. Finally, two reciprocal donor‐derived mechanisms cannot be excluded, though neither is testable retrospectively without paired donor follow‐up: exogenous alpha‐gal antigen introduced via a donor's recent meat ingestion or animal dander exposure, and passive transfer of alpha‐gal‐specific IgE from a donor with undiagnosed AGS, which could engage alpha‐gal epitopes from the recipient's own recent meat ingestion or, in AB or B recipients, histo‐blood group antigens on recipient cells.^31^ Despite these limitations, the findings have immediate quality improvement implications and support institutional workflow changes to improve AGS recognition during pretransfusion assessment and reduce exposure to higher‐risk plasma‐containing components in susceptible recipients.

In summary, we describe two patients with reactions consistent with TRAGS from the southeastern United States. Both patients had established AGS diagnoses in the medical record at the time of transfusion that were not recognized as transfusion risk factors, illustrating that documentation alone is insufficient without systems to translate that information into component selection decisions. Our retrospective analysis documents a corresponding institutional diagnostic gap spanning 5 years and 50 qualifying reactions, consistent with the broader healthcare system awareness gap that has accompanied the recent recognition of AGS as a transfusion risk. Transfusion medicine services in *Amblyomma americanum*‐endemic regions should consider incorporating AGS history into pre‐transfusion risk assessment, developing EMR‐based alerts for known AGS patients requiring plasma‐containing components, and including alpha‐gal IgE testing in the workup of unexplained ATRs in group O or A recipients following exposure to group B or AB plasma‐containing products. Future directions include the need for prospective study using standardized history ascertainment, predefined diagnostic criteria, and systematic alpha‐gal‐specific testing to better define the true frequency, clinical spectrum, and preventability of TRAGS.

## FUNDING INFORMATION

Dr. Stone receives funding for alpha‐gal related research from NIAID U01AI181927.

## CONFLICT OF INTEREST STATEMENT

The authors have disclosed no conflicts of interest. Dr. Jacobs is a member of the AABB Clinical Transfusion Medicine Committee; the views discussed in this article do not necessarily represent those of the AABB or the AABB CTMC.

## Data Availability

The data that support the findings of this study are available on request from the corresponding author. The data are not publicly available due to privacy or ethical restrictions.
